# Syndecan-1 as a prognostic biomarker in COVID-19 patients: a retrospective study of a Japanese cohort

**DOI:** 10.1186/s12959-024-00619-2

**Published:** 2024-06-21

**Authors:** Kiyohito Hayashi, Daisuke Koyama, Yoichi Hamazaki, Takamichi Kamiyama, Shingo Yamada, Miki Furukawa, Yoshinori Tanino, Yoko Shibata, Takayuki Ikezoe

**Affiliations:** 1 Department of Hematology, Fukushima Medical University, Fukushima, Fukushima 960-1295 Japan; 2 Department of Hematology, Iwaki City Medical Center, Iwaki, Fukushima Japan; 3 Department of Pediatric Surgery, Iwaki City Medical Center, Iwaki, Fukushima Japan; 4 R&D Center, Shino-Test Corporation, Sagamihara, Kanagawa Japan; 5 Division of Hematology, Kita-Fukushima Medical Center, Date, Fukushima Japan; 6 Department of Pulmonary Medicine, Fukushima Medical University, Fukushima, Fukushima Japan

**Keywords:** Syndecan-1, Coronavirus, Coronavirus disease 2019

## Abstract

**Background:**

The coronavirus disease 2019 (COVID-19) pandemic, caused by severe acute respiratory syndrome coronavirus 2 (SARS-CoV-2), has had a profound global impact, with millions of confirmed cases and deaths worldwide. While most cases are mild, a subset progresses to severe respiratory complications and death, with factors such as thromboembolism, age, and underlying health conditions increasing the risk. Vascular endothelial damage has been implicated in severe outcomes, but specific biomarkers remain elusive. This study investigated syndecan-1 (SDC-1), a marker of endothelial damage, as a potential prognostic factor for COVID-19, focusing on the Japanese population, which is known for its aging demographics and high prevalence of comorbidities.

**Methods:**

A multicenter retrospective study of COVID-19 patients in Fukushima Prefecture in Japan who were admitted between February 2020 and August 2021 was conducted. SDC-1 levels were measured along with other clinical and laboratory parameters. Outcomes including thrombosis, 28-day survival, and disease severity were assessed, and disease severity was categorized according to established guidelines.

**Results:**

SDC-1 levels were correlated with disease severity. Patients who died from COVID-19 had greater SDC-1 levels than survivors, and the area under the receiver operating characteristic curve (AUC) analysis suggested the potential of the SDC-1 level as a predictor of mortality (AUC 0.714). K‒M analysis also revealed a significant difference in survival based on an SDC-1 cutoff of 10.65 ng/mL.

**Discussion:**

This study suggested that SDC-1 may serve as a valuable biomarker for assessing COVID-19 severity and predicting mortality within 28 days of hospitalization, particularly in the Japanese population. However, further investigations are required to assess longitudinal changes in SDC-1 levels, validate its predictive value for long-term survival, and consider its applicability to new viral variants.

**Conclusions:**

SDC-1 is emerging as a potential biomarker for assessing the severity and life expectancy of COVID-19 in the Japanese population, offering promise for improved risk stratification and patient management in the ongoing fight against the virus.

## Background

COVID-19, caused by severe acute respiratory syndrome coronavirus 2 (SARS-CoV-2), has had an enormous impact on global health and the economy [1, 2]. More than 767 million cases have been confirmed worldwide, and 6.9 million people have died as of May 2023 [3]. Although the World Health Organization (WHO) declared the end of the COVID-19 state of emergency in May 2023, the threat of infection by mutant strains remains.

Most cases of COVID-19 are mild or asymptomatic, but a small percentage of those who develop pneumonia progress to acute respiratory distress syndrome (ARDS) and require mechanical ventilation. The leading cause of death in COVID-19 patients is respiratory failure due to ARDS [4]. Although respiratory failure is the most common cause of death, mortality has been found to be greater in patients complicated by thromboembolism [5]. Mortality is also known to be greater in elderly individuals and in patients with underlying medical conditions such as hypertension, ischemic heart disease (IHD), and diabetes mellitus (DM) [5]. Numerous studies and meta-analyses have been conducted on biomarkers to determine disease severity and predict prognosis to identify target populations that should be heavily treated to improve survival [6–10].

Vascular endothelial damage is considered a risk factor for thrombosis, but there is no consensus on biomarkers suggestive of vascular endothelial damage in COVID-19 patients. A candidate biomarker for vascular endothelial damage is syndecan-1 (SDC-1), a core protein of heparan sulfate proteoglycans expressed on endothelial cells. SDC-1 forms glycocalyx that plays a role in regulating vascular permeability and preventing thrombus formation and leukocyte adhesion [11]. Previous studies found that circulating SDC-1 is an important marker of glycocalyx degradation associated with severity and prognosis in cardiovascular disease (CVD) [11–14]. Under inflammatory conditions, the accumulation of proteases, including matrix metalloproteinases, thrombin, and plasmin, accelerates the shedding of SDC-1 from the endothelial surface [15, 16]. Several indicators have demonstrated that serum SDC-1 levels are significantly correlated with the Sequential Organ Failure Assessment (SOFA) score in patients with sepsis [17–19], and that non-survivors of sepsis have significantly higher SDC-1 levels than survivors. These findings suggest the potential utility of SDC-1 as a biomarker linking endotheliopathy to organ failure [19–21].

Japan has an aging society with a high proportion of elderly people and a high prevalence of underlying diseases such as hypertension and diabetes. However, during the early stages of the COVID-19 pandemic, the mortality rate in Japan was lower than that in other countries [22].

The purpose of this study was to investigate whether there is an association between SDC-1 levels and disease severity and life expectancy in Japanese COVID-19 patients.

## Methods

### Study location and patients

This was a multicenter retrospective study conducted at Fukushima Medical University Hospital and Iwaki City Medical Center. The participants were all COVID-19 patients who were admitted to Fukushima Medical University Hospital and Iwaki City Medical Center between February 2020 and August 2021.

### Severity classification of COVID-19

COVID-19 cases are generally divided into four severity categories based on the guidelines of the Ministry of Health, Labor and Welfare in Japan [23]. Mild cases were defined as cases with mild clinical symptoms but no evidence of pneumonia on CT scan. Moderate cases 1 were defined as cases with mild respiratory symptoms and mild hypoxemia with pneumonia but no evidence of oxygen requirements. Moderate cases 2 were defined as cases with SpO2 less than 93% with oxygen required. Severe cases were defined as cases with intensive care unit management and those requiring mechanical ventilation or extracorporeal membrane oxygenation (ECMO) support. The WHO classification offers more detailed criteria for each severity level, especially concerning respiratory symptoms and oxygen saturation. The WHO classification of mild, moderate, severe, and critical roughly corresponds to the MHLW’s classification of disease severity as mild, moderate-1, moderate-2, and severe.

### Blood samples

Blood samples were collected on the day of admission or the day after admission. White blood cell count, neutrophil count, lymphocyte count, hemoglobin, platelet count, LD, PT-INR, APTT, D-dimer, CRP, total bilirubin, ALT, AST, and creatinine were measured. SDC-1 was measured by an enzyme-linked immunosorbent assay established by the R&D Center, Shino-Test Corporation, using residual serum from blood tests [24]. Serum was separated from blood samples collected using blood collection tubes containing a serum separator.

### Data collection

Other data included age, sex, body mass index (BMI), smoking history, underlying medical conditions, laboratory data, intensive care unit admission, use of mechanical ventilation or ECMO, and outcome. The associations of these outcomes with thrombosis incidence, disease severity, and survival at 28 days after admission were analyzed.

### Statistical analysis

The data are presented as the median, 25th-75th percentile or number (%). Statistical analysis was performed with EZR. Wilcoxon’s rank-sum test, chi-square test, Student’s t test, and Kruskal-Wallis test were used for between-group comparisons, as appropriate. Multivariate analysis was also performed using logistic regression analysis. The usefulness of each parameter in predicting outcome was assessed by area under the receiver operating characteristic (ROC) curve (AUC) analysis; a *P* value < 0.05 was considered to indicate statistical significance.

### Study approval

This study was conducted in accordance with the Declaration of Helsinki and was approved by the General Ethics Committee of Fukushima Medical University (Approval Review Number: General 2021-056). This retrospective observational study was conducted, and all patients were clearly informed that the study was a clinical study and were given the opportunity to opt out, as well as consent to participate in the study.

## Results

### Patient characteristics

A total of 381 COVID-19 patients were included in this study. Table 1 shows the clinical characteristics of these patients. A total of 118, 108, 90, or 65 patients were classified as having mild, moderate 1, moderate 2, or severe disease, respectively. As in previous reports, age and BMI tended to increase with disease severity [25, 26]. In addition, males had higher disease severity on average than females. A greater percentage of patients with more severe disease had a history of smoking, hypertension, IHD, COPD, or DM. The more severely ill patients were admitted to the ICU and required mechanical ventilation and ECMO support. Two patients (2.2%) in the moderately ill group were also critically ill on admission and received ECMO support. Twenty-two patients died while hospitalized, 15 of whom died within 28 days of admission. White blood cell count, serum LD, CRP, and serum creatinine levels increased with disease severity. Pulmonary function tests were not performed during hospitalization for COVID-19 patients because of efforts to prevent infection to avoid the spread of pathogens in the hospital.

**Table 1:** The clinical characteristics of the COVID-19 patients

	Mild (*n* = 119)	Moderate-1 (*n* = 105)	Moderate-2 (*n* = 90)	Severe (*n* = 65)	*P* values
Age (median [IQR], year)	36 [24.0, 60.5]	59[47.0, 70.0]	65 [53.5, 74.5]	66 [59.0, 73.0]	< 0.001
Sex (male, %)	55 (46.2)	55 (52.4)	61 (67.8)	46 (70.8)	0.001
BMI (median [IQR], kg/m^2^)	22.9 [19.7, 26.0]	24.0 [22.0, 27.2]	25.6 [23.4, 29.7]	26.3 [24.6, 30.9]	< 0.001
Smoking (%)	41 (36.3)	44 (44.4)	55 (63.2)	28 (50.0)	0.002
Comorbidity					
Hypertension (%)	26 (21.8)	41 (39.0)	50 (55.6)	41 (63.1)	< 0.001
DM (%)	14 (11.8)	21 (20.0)	24 (26.7)	24 (36.9)	0.001
COPD (%)	0 (0.0)	0 (0.0)	7 (7.8)	5 (7.7)	< 0.001
IHD (%)	2 (1.7)	3 (2.9)	8 (8.9)	3 (4.6)	0.064
Asthma (%)	13 (10.9)	4 (3.8)	5 (5.6)	4 (6.2)	0.18
Blood biomarkers					
White blood cell (median [IQR], 10^3^/µL)	4.80 [3.65, 6.05]	4.70 [3.90, 6.10]	7.15 [5.12, 9.65]	7.00 [5.40, 9.80]	< 0.001
Neutrophils (median [IQR], 10^3^/µL)	2.80 [1.90, 3.60]	3.20 [2.40, 4.30]	5.55 [3.70, 7.70]	5.40 [3.80, 8.60]	< 0.001
Lymphocytes (median [IQR], 10^3^/µL)	1.40 [1.10, 1.80]	1.00 [0.80, 1.50]	0.80 [0.50, 1.08]	0.90 [0.60, 1.20]	< 0.001
Hemoglobin (median [IQR], g/dL)	14.1 [12.6, 15.7]	13.9 [12.2, 14.9]	13.5 [11.9, 14.8]	14.4 [12.7, 15.4]	0.027
Platelet (median [IQR], 10^3^/µL)	219.0 [182.5, 267.5]	193.0 [159.0, 239.0]	200.5 [156.2, 249.5]	189.0 [161.0, 231.0]	0.002
Total Bilirubin (median [IQR], mg/dL)	0.50 [0.40, 0.67]	0.50 [0.40, 0.60]	0.60 [0.40, 0.70]	0.60 [0.40, 0.80]	0.018
LD (median [IQR], U/L)	189.0 [167.0, 216.5]	228.0 [199.0, 268.0]	319.0 [273.2, 422.2]	322.0 [247.0, 480.0]	< 0.001
Creatinine (median [IQR], mg/dL)	0.71 [0.58, 0.86]	0.76 [0.63, 0.96]	0.84 [0.68, 0.97]	0.84 [0.71, 1.02]	< 0.001
CRP (median [IQR], mg/dL)	0.14 [0.05, 0.40]	1.26 [0.40, 4.18]	5.14 [2.86, 9.05]	4.02 [1.67, 9.17]	< 0.001
D-dimer (median [IQR], µg/mL)	0.50 [0.50, 0.65]	0.50 [0.50, 1.20]	1.05 [0.50, 1.87]	0.80 [0.50, 1.80]	< 0.001
SDC-1 (median [IQR], ng/mL)	4.90 [3.70, 7.00]	6.30 [4.40, 11.90]	10.15 [6.03, 18.25]	10.30 [5.40, 25.40]	< 0.001
ICU (%)	0 (0.0)	0 (0.0)	0 (0.0)	54 (96.4)	< 0.001
ECMO (%)	0 (0.0)	0 (0.0)	2 (2.2)	5 (7.7)	0.001
Hospital mortality within 28days (%)	0 (0.0)	1 (1.0)	4 (4.4)	10 (15.4)	< 0.001

IQR: interquartile range, BMI: body mass index, DM: diabetes mellitus, COPD: chronic obstructive pulmonary disease, IHD: ischemic heart disease, LD: lactate dehydrogenase, CRP: C-reactive protein, SDC-1: syndecan-1, ICU: intensive care unit, ECMO: extracorporeal membrane oxygenation

### Prediction of disease severity and hospital mortality using serum SDC-1 levels

**Fig. 1:**
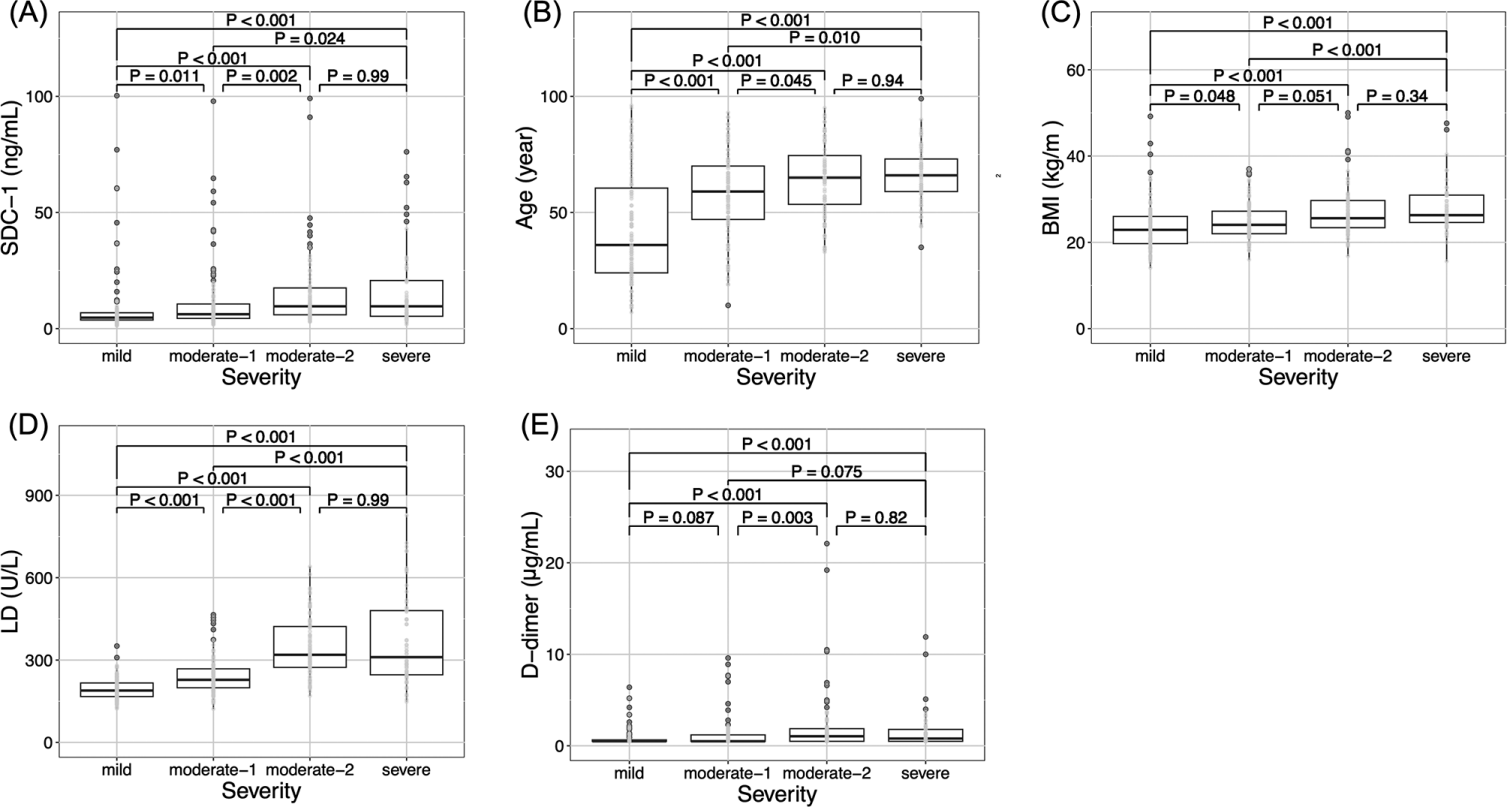
Factors associated with COVID-19 severity. (**A**) SDC-1; (**B**) age; (**C**) BMI; (**D**) LD; (**E**) D-dimer. The center bold line is the median value; the bottoms and tops of the boxes represent the 25th and 75th percentiles, respectively; and the whiskers are 95% confidence intervals. SDC-1, syndecan-1; BMI, body mass index; LD, lactate dehydrogenase

**Fig. 2:**
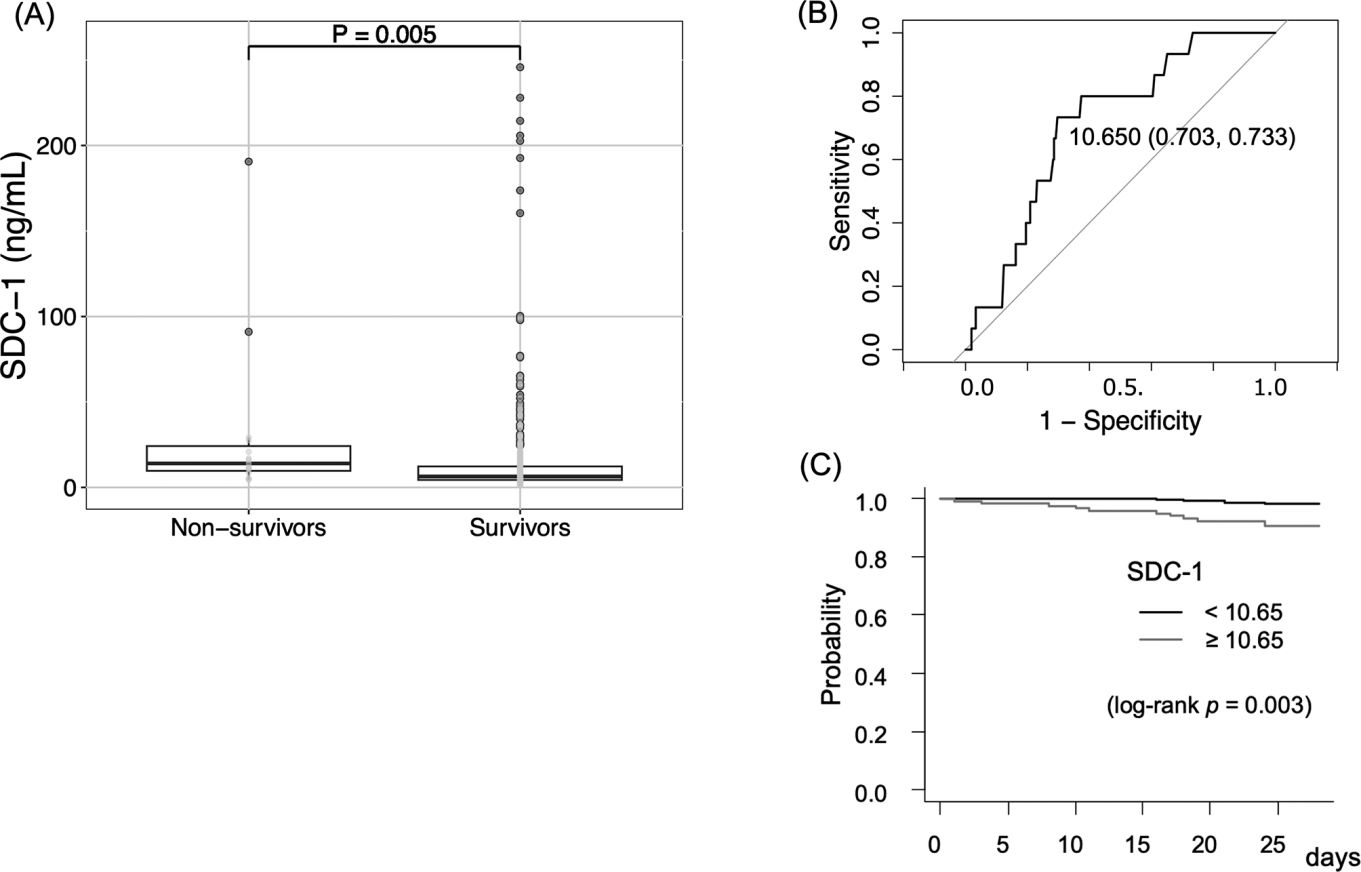
Prediction of in-hospital mortality using serum SDC-1 levels. (**A**) Serum SDC-1 levels in survivors and nonsurvivors. The lines in the graph indicate the medians with interquartile ranges. The differences between the survivors and nonsurvivors were analyzed using the Mann-Whitney U test (*P* = 0.005). (**B**) Receiver operating characteristic (ROC) curve analysis of the ability of serum SDC-1 levels to predict in-hospital mortality. The area under the ROC curve (AUC) for predicting hospital mortality was 0.714. (**C**) Survival rate according to the Kaplan-Meier method in which patients with SDC-1 were divided into two groups according to the cutoff point. Survival analysis by the Kaplan-Meier method revealed a significant difference in the SDC-1 value (*p* = 0.003). SDC-1, syndecan-1

SDC-1 values significantly increased with disease severity (Kruskal-Wallis test, *p* < 0.001) (Fig. 1). The median level of SDC-1 in healthy volunteers (*n* = 8, median age 32 years) was 2.6 ng/mL (range: 1.8–3.8 ng/mL), which was significantly lower than the mild case of the COVID-19 patients (*p* = 0.002, data not shown). The patients who died within 28 days of hospitalization and those who survived were divided into two groups, and a comparison between the two groups using SDC-1 values revealed a significant difference (Mann-Whitney U test, *p* = 0.005) (Fig. 2). ROC curve analysis revealed that the AUC for predicting mortality in COVID-19 patients within 28 days of hospitalization for serum SDC-1 was 0.714 (95% CI, sensitivity 73.3%, specificity 70.3%), with an optimal cutoff value of 10.65 ng/mL (Fig. 2).

### Age and SDC-1 are predictive of poor prognosis in COVID-19 patients

COVID-19 patients were divided into two groups using an SDC-1 cutoff of 10.65 ng/mL. The Kaplan-Meier survival test showed a significant difference between the two groups (log-rank *p* = 0.003; Fig. 2). The group with SDC-1 levels ≥ 10.65 ng/mL had a significantly greater mortality rate within 28 days of hospitalization (HR 6.2; 95% CI 2.0-19.7; *p* = 0.001; Table 2). Cox proportional hazards regression analysis was performed with older age, BMI > 30 kg/m^2^, diabetes status, CVD status, and SDC-1 status as independent variables. Age and SDC-1 were found to be statistically significant. An SDC-1 concentration ≥ 10.65 ng/mL had a hazard ratio of 7.3 (95% confidence interval of 1.50–34.8, *p* = 0.012). According to the multivariate analysis, BMI > 30 kg/m^2^, diabetes status, and heart disease status were not significantly different between the two groups (Table 2).

**Table 2:** Cox’s proportional hazards regression

Characteristics	*n* = 379, (%)	Univariate				Multivariate		
		HR	95% CI	*P*		HR	95% CI	*P*
Age, year								
≧ 65	145 (38)	23.7	3.1-180.2	0.002		12.9	1.5–110.0	0.018
< 65	234 (62)	1						
BMI, kg/m^2^								
≧ 30	60 (16)	2.1	0.5–8.1	0.28		3.1	0.7–12.7	0.11
< 30	291 (77)	1						
Diabetes mellitus								
yes	83 (22)	1.3	0.4–4.1	0.63		0.6	0.1–2.8	0.52
no	296 (78)	1						
Hypertension								
yes	158 (42)	3.9	1.2–12.3	0.018		2.2	0.4–11.1	0.3
no	221 (58)	1						
Ischemic heart disease								
yes	16 (4)	1.5	0.2–12.1	0.65		1.3	0.1–11.0	0.79
no	363 (96)	1						
SDC-1, ng/mL								
≧ 10.7	119 (31)	6.2	2.0-19.7	0.001		7.3	1.5–34.8	0.012
< 10.7	260 (69)	1						

BMI: body mass index, SDC-1: syndecan-1, HR: hazard ratio, CI: confidence interval

## Discussion

In this study, we focused on SDC-1, a component of the glycan chain expressed on the surface of the vascular endothelium, as a potential biomarker for assessing the severity of COVID-19 since endothelialitis is thought to be involved in the pathogenesis of severe COVID-19. We investigated the relationships between SDC-1 levels, COVID-19 disease severity, and mortality within 28 days of hospitalization in Japanese COVID-19 patients. Our ROC analysis revealed that SDC-1 levels increase with disease severity, suggesting that SDC-1 may serve as a valuable prognostic marker. These results are consistent with previous studies that compared SDC-1 levels between surviving and deceased COVID-19 patients in the Middle East and China and found that SDC-1 levels were significantly greater in patients who succumbed to COVID-19 than in survivors [27, 28]. In addition, compared with those in non-ICU patients, SDC-1 levels on the first day of admission were significantly elevated in ICU patients [27]. ROC analysis revealed that the AUC for the ability of the SDC-1 concentration to predict ICU admission for COVID-19 patients at admission was 0.705. Furthermore, in predicting COVID-19 mortality, ROC analysis revealed that the optimal cutoff value of SDC-1 for distinguishing nonsurvivors from survivors (813.8 ng/mL) had a sensitivity of 68.6%, a specificity of 78.6%, and an AUC of 0.783 (95% confidence interval [CI] 0.647–0.918, *P* = 0.002) [28]. Even if the disease is classified as hypothetically mild in terms of severity, a high SDC-1 value might prompt consideration for more intensive treatment to prevent progression to severe disease. Elevated SDC-1 levels may also indicate vascular endothelial damage, and addressing such damage through treatment could potentially improve prognosis.

Other reports and meta-analyses have been published on other biomarkers of disease severity [6, 7], showing a significant association between lymphocyte count, CRP, procalcitonin, LD, and D-dimer and COVID-19 severity [6].

Elevated serum levels of SDC-1 indicate the loss of endothelial glycans, the glycocalyx, which play a role in maintaining vascular integrity and coagulation homeostasis [29]. The exact mechanism underlying the elevated SDC-1 levels in COVID-19 patients remains unclear. The shedding and degradation of the glycocalyx are observed in other disease states, including sepsis and ARDS [30]. Inflammation-induced activation of metalloproteinases, heparanases, and hyaluronidases is thought to play a role in glycocalyx shedding and/or degradation [30].

As a result of endothelial damage, a hypercoagulable state is induced. We assumed that SDC-1 levels would correlate with D-dimer levels. Contrary to our expectation, no correlation was observed between these levels (data not shown), suggesting that COVID-19-associated coagulopathy is not simply a result of endothelialitis. The exaggerated production of cytokine-induced tissue factors on inflammatory cells and endothelial cells, as well as neutrophil extracellular traps released by activated neutrophils, may collaboratively play a role in this condition.

Our study showed that disease severity was significantly greater in patients with DM, hypertension, and IHD than in those without these comorbidities (Table 1). These comorbidities are strongly associated with vascular endothelial damage [31, 32]. Therefore, we hypothesized that patients with these comorbidities would also have higher SDC-1 levels than those without these comorbidities. However, contrary to our expectation, we did not find significant group differences in the SDC-1 values (data not shown), suggesting that vascular endothelial damage in COVID-19 patients may be different from that in patients with DM, hypertension, and IHD, which are often complicated by atherosclerosis. Although DM and CVD are risk factors for disease severity [33, 34], they may not serve as prognostic predictors (Table 2).

This study has several limitations. First, SDC-1 levels were measured only at admission, and changes over time are unknown. Therefore, it is possible that SDC-1 levels may have increased over time in deceased patients. Second, further validation is necessary to confirm the validity of the SDC-1 as a predictor of long-term survival, as some patients died after 28 days of hospitalization. Third, the subjects in this study were cases from the period when alpha and delta variants were prevalent, and it remains uncertain whether similar results can be obtained for cases involving other variants.

## Conclusions

SDC-1 may be a promising biomarker for the prediction of disease severity and life expectancy in Japanese COVID-19 patients.

## Data Availability

The datasets obtained and analyzed in the current study are available from the corresponding author on reasonable request.

## Abbreviations

COVID-19: Coronavirus disease 2019
SARS-CoV-2: Severe acute respiratory syndrome coronavirus
SDC-1: Syndecan-1
ELISA: Enzyme-linked immunosorbent assay
ROC: Receiver operating characteristic
AUC: Area under curve
FDP: Fibrinogen/fibrin degradation products
PT-INR: Prothrombin time international normalized ratio
CRP: C-reactive protein
SpO2: saturation of percutaneous oxygen
ECMO: Extracorporeal membrane oxygenation
